# A rare serious case of retroperitoneal paraganglioma misdiagnosed as duodenal gastrointestinal stromal tumor: a case report

**DOI:** 10.1186/s12893-020-00712-z

**Published:** 2020-03-16

**Authors:** Schauki Mahmoud, Maissam Salami, Hosam Salman

**Affiliations:** 1 Department of General Surgery, Albassel Hospital, Tartous, Syrian Arab Republic; 2 Department of Anaesthesiology, Albassel Hospital, Tartous, Syrian Arab Republic; 3 Department of Histopathology, Albassel Hospital, Tartous, Syrian Arab Republic

**Keywords:** Paraganglioma, Retroperitoneal mass, Hypertensive crisis, Catecholamine secreting tumor, GIST

## Abstract

**Background:**

Pheochromocytoma (PCC) and Paraganglioma (PGL) are rare neuroendocrine neoplasms. These tumors harbour disastrous consequences during surgery due to catecholamine hypersecretion if they are undiagnosed or prepared inadequately preoperatively.

**Case presentation:**

A 41- year- old lady presented with mild left flank discomfort. She had experienced recurrent anxiety attacks accompanied by palpitations and headache which were managed previously as panic attacks. Radiologic investigations showed a retroperitoneal mass that located anteromedial to the left kidney, separated from the left adrenal gland and adherent to the 4th duodenal segment. During admission, her vital signs showed slight elevation of blood pressure (140\90–160\110) mmHg, thus 24-h urine metanephrine and normetanephrine were requested and the results revealed normal values. Upper gastrointestinal endoscopy failed to pass beyond the 3th duodenal segment and showed no pathologic evidence. According to her findings, a diagnosis of duodenal gastrointestinal stromal tumor (GIST) was suspected. During laparotomy, crises of hypertension and tachycardia followed by severe hypotension made the resection of the misdiagnosed mass very tricky. Immunohistochemical staining studies confirmed the diagnosis of paraganglioma.

**Conclusion:**

Paraganglioma is a life threatening disease and should always be considered as a differential diagnosis of asymptomatic retroperitoneal mass. The aim of our study is to present a challenging case of an undiagnosed retroperitoneal paraganglioma and to alarm our colleagues from such troubles.

## Background

Pheochromocytoma (PCC) and paraganglioma (PGL) are rare neuroendocrine tumors of chromaffin cells which secrete catecholamines from adrenal medulla (80–90%) and extra-adrenal paraganglia (10–20%) respectively [1].

The incidence of pheochromocytoma and paraganglioma (PPGL) is estimated to be about 0.2–1\100,000 [1].

Most of PGLs (75%) are located retroperitoneal in the organs of Zuckerkandl. PGLs could be symptomatic due to catecholamine secretion or asymptomatic. Asymptomatic PGLs during abdominal imaging could easily mimic other retroperitoneal masses as GIST.

PGLs tend to be sporadic and could be hereditary in approximately 30% of cases including germline mutations in Von Hippel-Lindau syndrome, succinate dehydrogenase subunits SDHx, RET, neurofibromatosis type 1 and multiple endocrine neoplasia (MEN2) [2–4]. There is obvious correlation between SDHB, SDHD mutations and PGLs [4].

During surgery, undiagnosed PGL or inadequate preoperative preparation can be life threatening due to hypertensive crisis followed by hypotension and cardiac arrhythmia. We hereby present a case of retroperitoneal PGL which was misdiagnosed as duodenal GIST.

## Case presentation

A 41-year- old lady presented with a history of mild left flank discomfort and mild hyperglycemia. She had experienced recurrent anxiety attacks accompanied by palpitations and headache. She denied any history of hypertension. She was diagnosed 3 years ago as a panic disorder patient and was treated with fluoxetine and chlorpromazine. She had a history of uncomplicated three deliveries and tonsillectomy.

Physical examination showed slightly pale patient, pulse rate about (90–110) beat per minute (bpm) and blood pressure (BP) about (140\90–160\110) mmHg. Mild tenderness in the left hypochondriac area was noticed.

Laboratory tests results are included in (Table 1).

**Table 1:** Admission work-up

White blood cell count 8700 mm3	Hemoglobin 10.1 g/dL	Platelet 210,000 mm3	Glucose 165 mg/dL
Urea 17 mg/dL	Creatinine 0.5 mg/dL	Potassium 4.3 mmol/L	Sodium 136 mmol/L
ALT 13 U/L	AST 11 U/L	TSH 0.45ulU/ml (0.25–5)	Free T3 236 pg/dL (140–440)
Free T4 0.95 ng/dL (0.8–2)	T.Prot 6.7 g/dL	ALB 3.6 g/dL	24-h urine metanephrine 55 mg/24 h (36–190)
24-h urine normetanephrine 190 mg/24 h (35–482)			

**Fig. 1:**
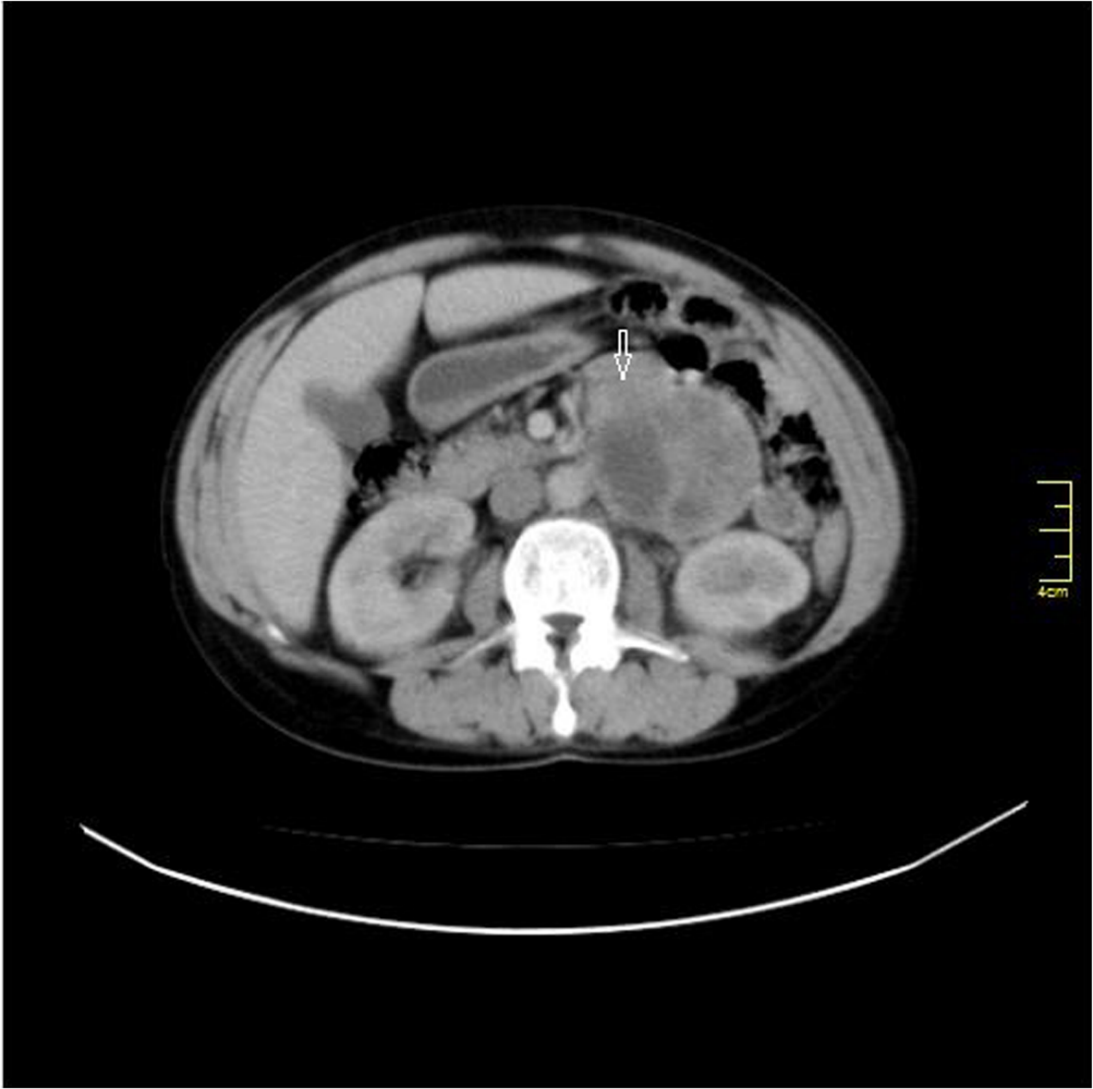
Axial abdomen C.T with I. V contrast shows 9 × 7.3 × 6.5 cm well defined heterogeneous retroperitoneal mass with peripheral enhancement and central necrosis mimic 4th segment duodenal GIST (white arrow)

**Fig. 2:**
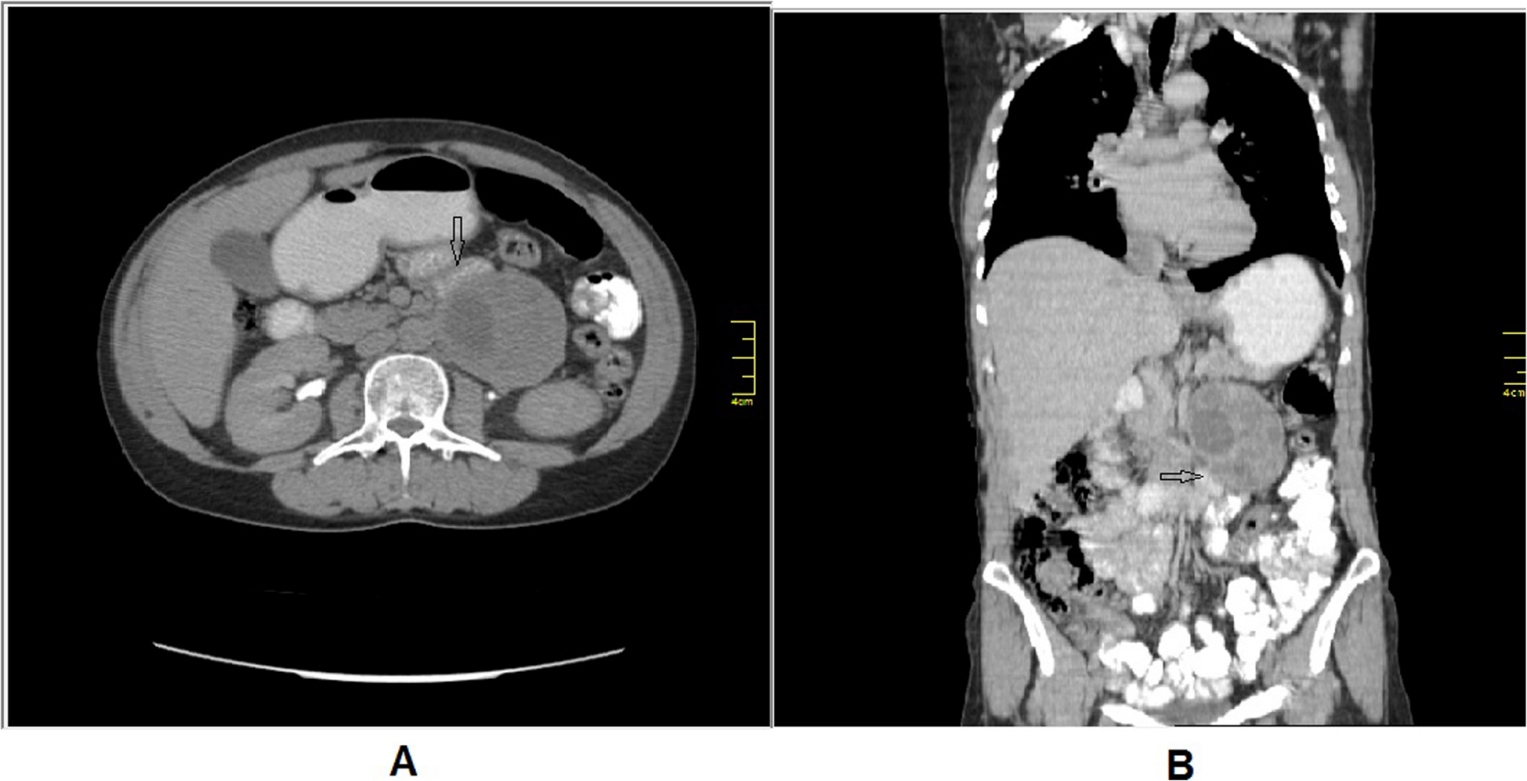
C.T abdomen with I. V and oral contrast: (A) axial section, (B) coronal section demonstrates the relationship between the mass and the 4th duodenal segment (black arrow)

**Fig. 3:**
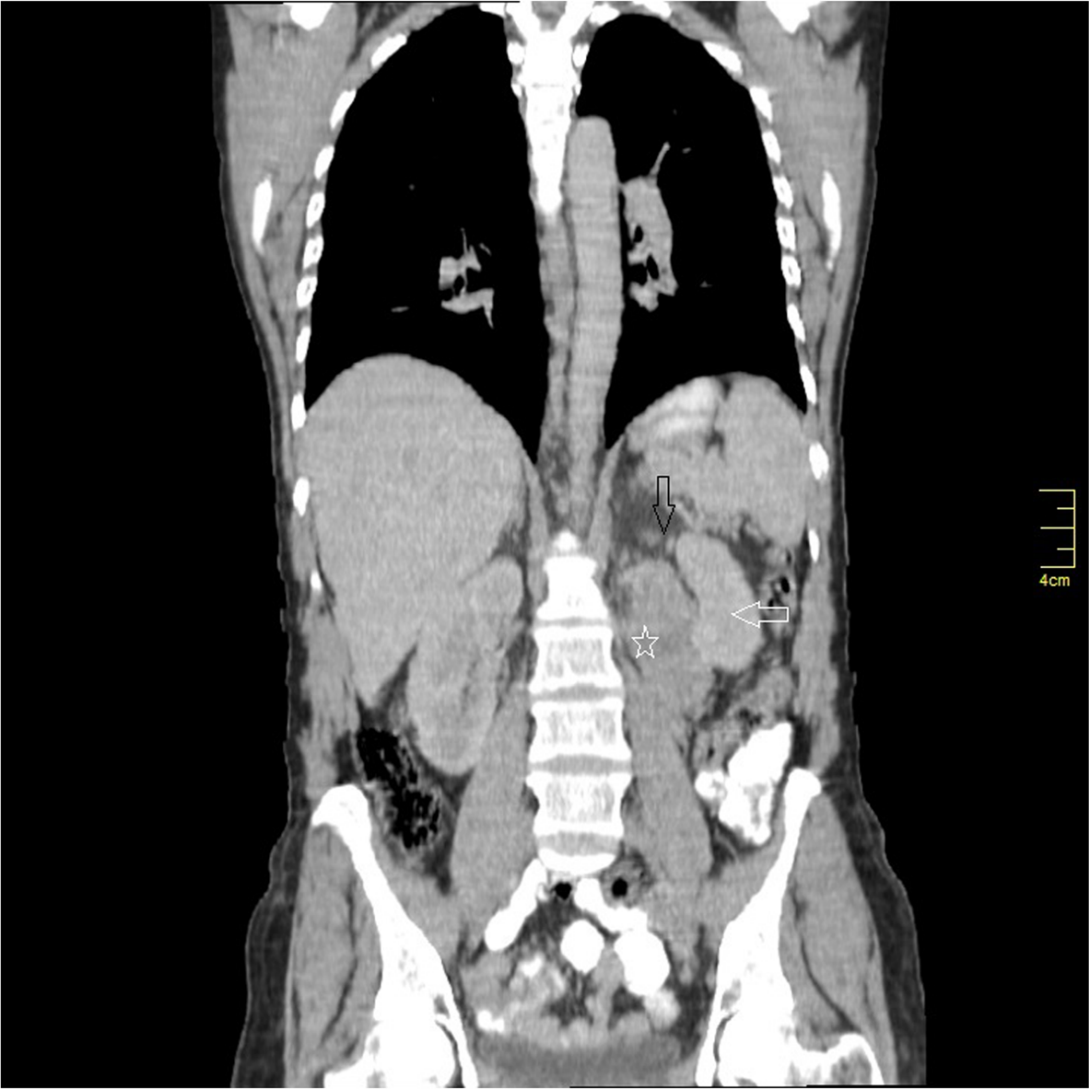
Coronal section C.T demonstrates the relationship between the mass (star), the left kidney (white arrow) and the left adrenal gland (black arrow)

Computed tomography (C.T) with intravenous and oral contrast demonstrated a well defined heterogeneous retroperitoneal mass 9 × 7.3 × 6.5 cm containing a necrotic centre with well peripheral enhancement. The mass was located anteromedial to the left kidney and separated from the left adrenal gland, anterolateral to the aorta and connected with the 4th duodenal segment. No evidence of lymph node enlargement or metastatic lesion was noticed (Figs 1, 2, 3). These findings raised a differential diagnosis of duodenal GIST.

Because of the presence of tachycardia attacks, mild hyperglycemia and newly discovered hypertension, we requested 24-h urine metanephrine and normetanephrine to rule out hormonally functioning mass. The results were within normal limits including thyroid function tests (Table 1).

Due to the lack of our facilities, upper gastrointestinal endoscopy failed to pass beyond the 3th segment of the duodenum and was normal.

Because of the unspecific symptoms of our patient, the C.T findings, the laboratory investigations (anemia and normal hormonal tests) and the absence of histological biopsy, we diagnosed the mass primarily as duodenal GIST.

The patient was scheduled for laparotomy to resect the suspected duodenal GIST.

Preoperative preparation included anti hyperglycaemic medication (Metformin 500 mg PO twice daily) and anti hypertensive medications (Prazosin 5 mg PO twice daily, Diltiazem 60 mg PO every 8 h). Fourteen days later, the patient was readmitted for laparotomy.

**Fig. 4:**
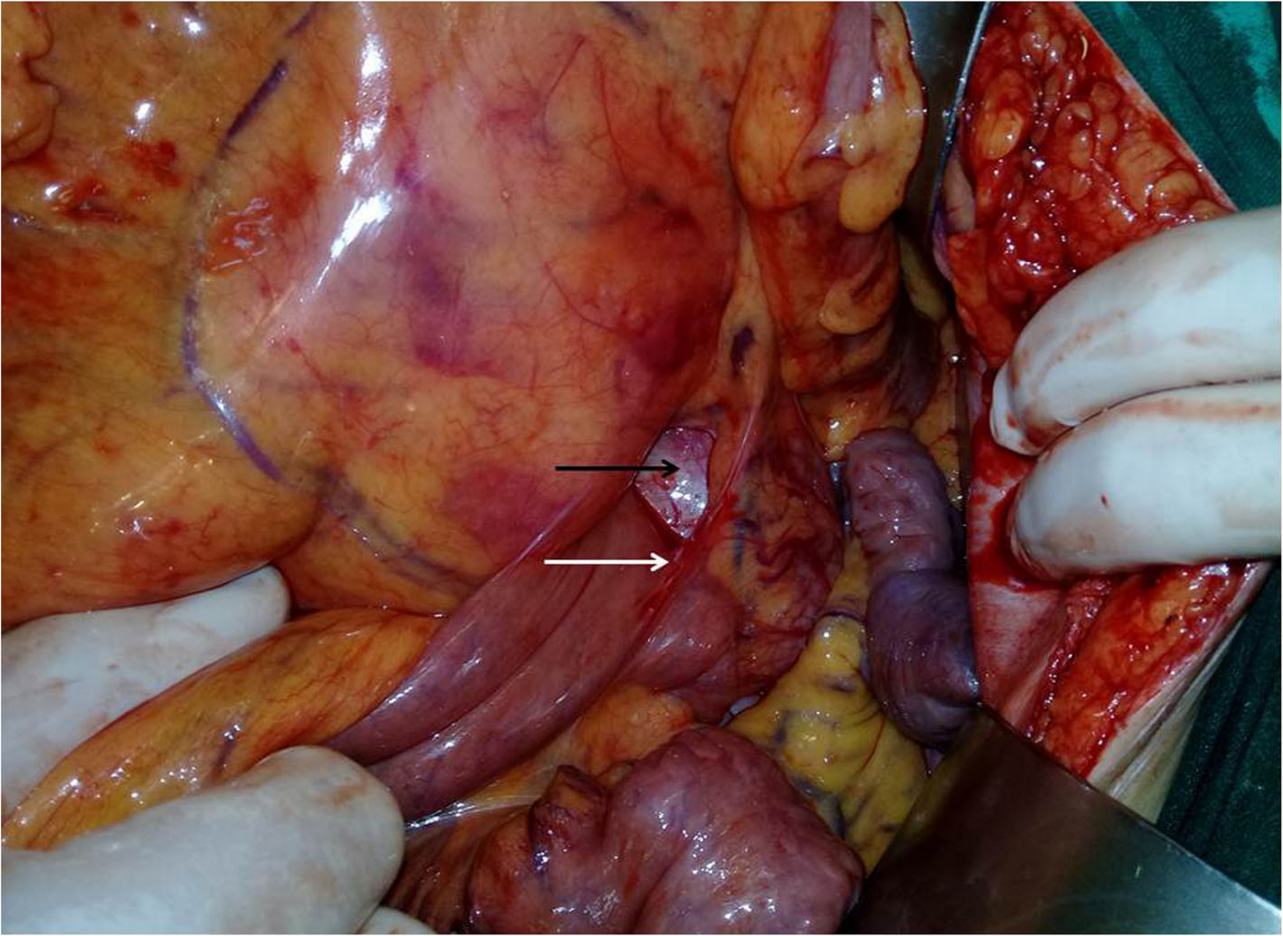
The mass (black arrow) was paravertebral retroperitoneal adherent to the 4th segment of the duodenum and Treitz ligament (white arrow).

**Fig. 5:**
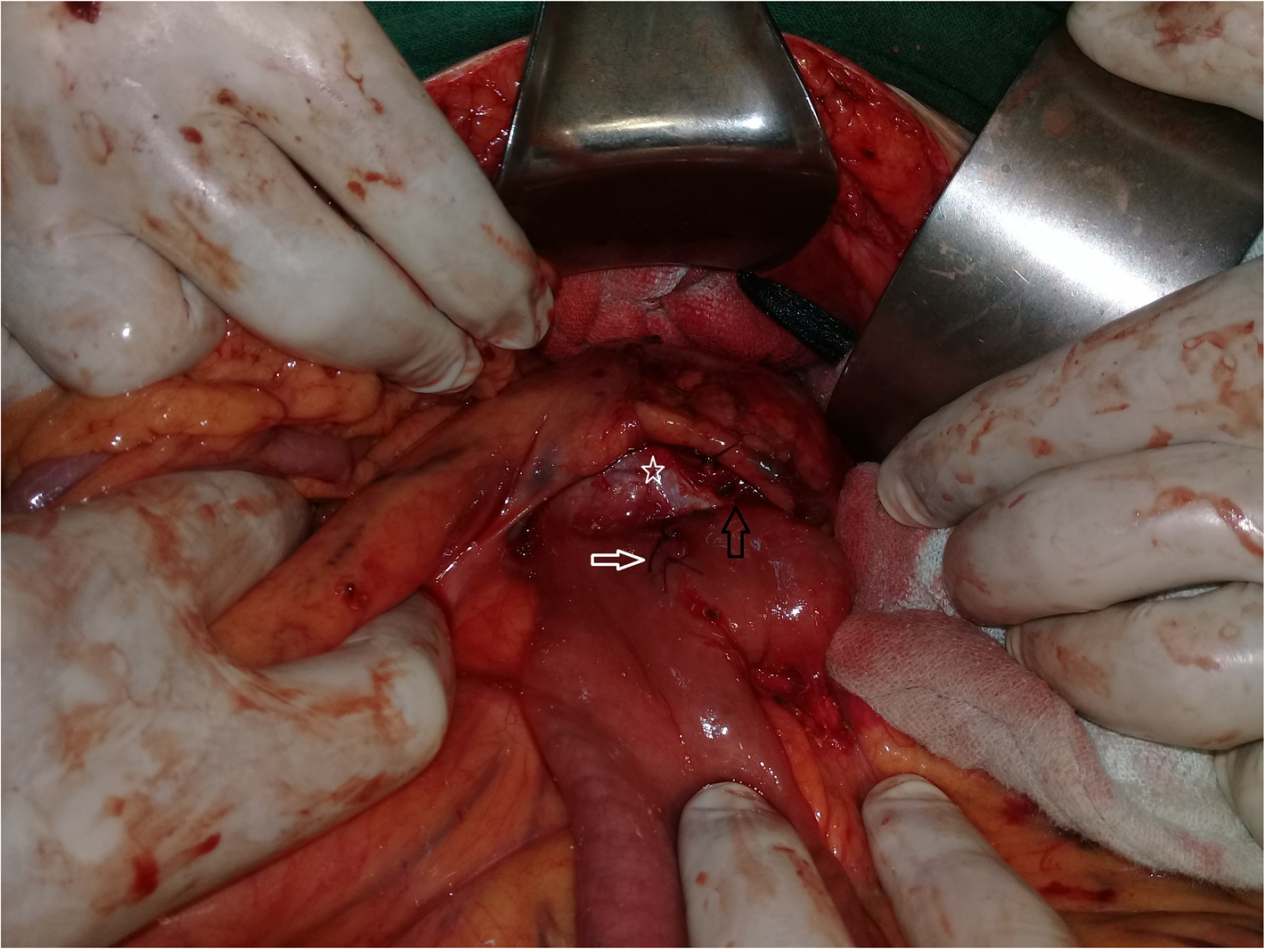
The tumor (star) shows neovascularisation (white arrow) from the duodeno-jejunal junction with strict adhesion with the 4th portion of the duodenum without invasion (black arrow).

**Fig. 6:**
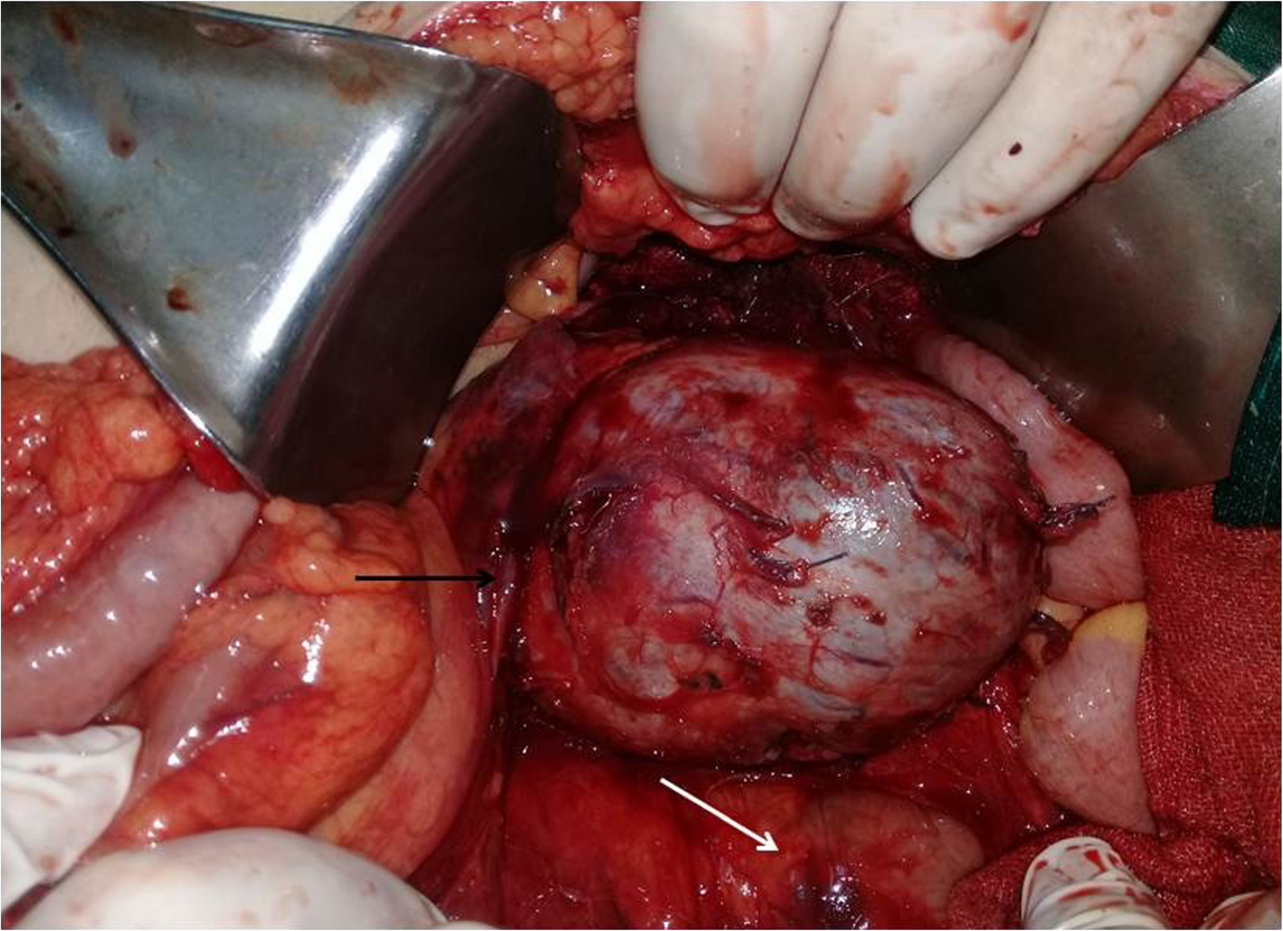
The relationship between the hypervascularized mass, the aorta (white arrow) and the left renal vein (black arrow).

**Fig. 7:**
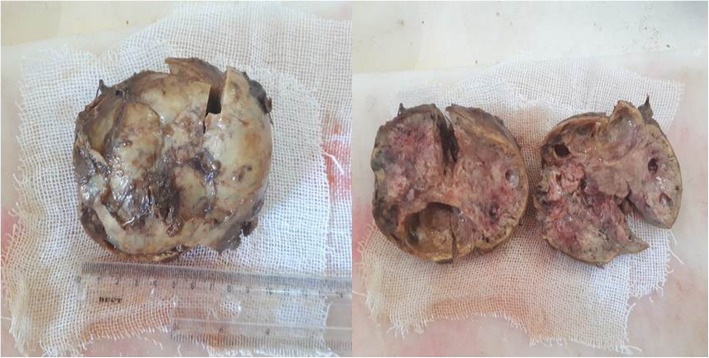
Gross pathology: Well-circumscribed mass with lobular red-brown appearance and necrosis.

**Fig. 8:**
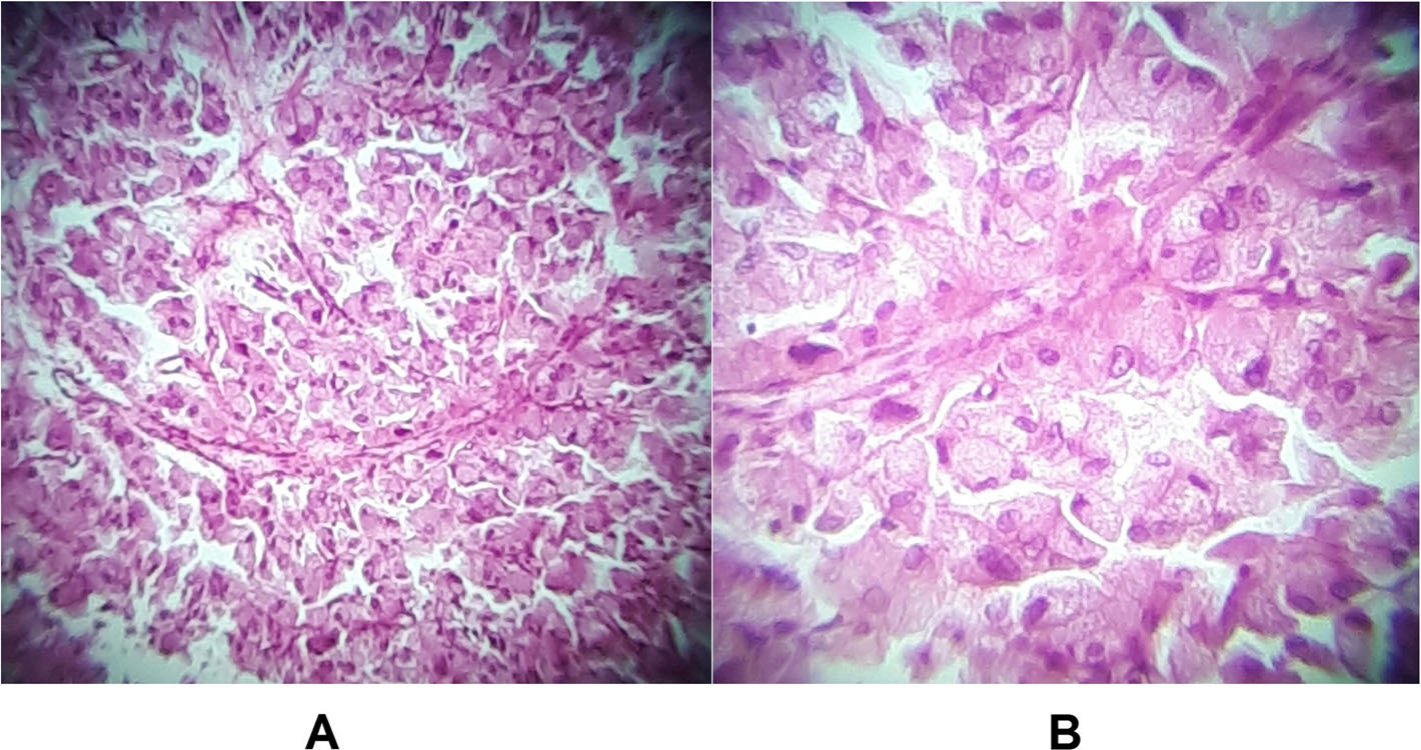
**a,b** H&E stain: Nests of round to polygonal tumor cells (Zellballen pattern), with central nuclei, nuclear atypia and eosinophilic cytoplasm which predicates the diagnosis of paraganglioma.

**Fig. 9:**
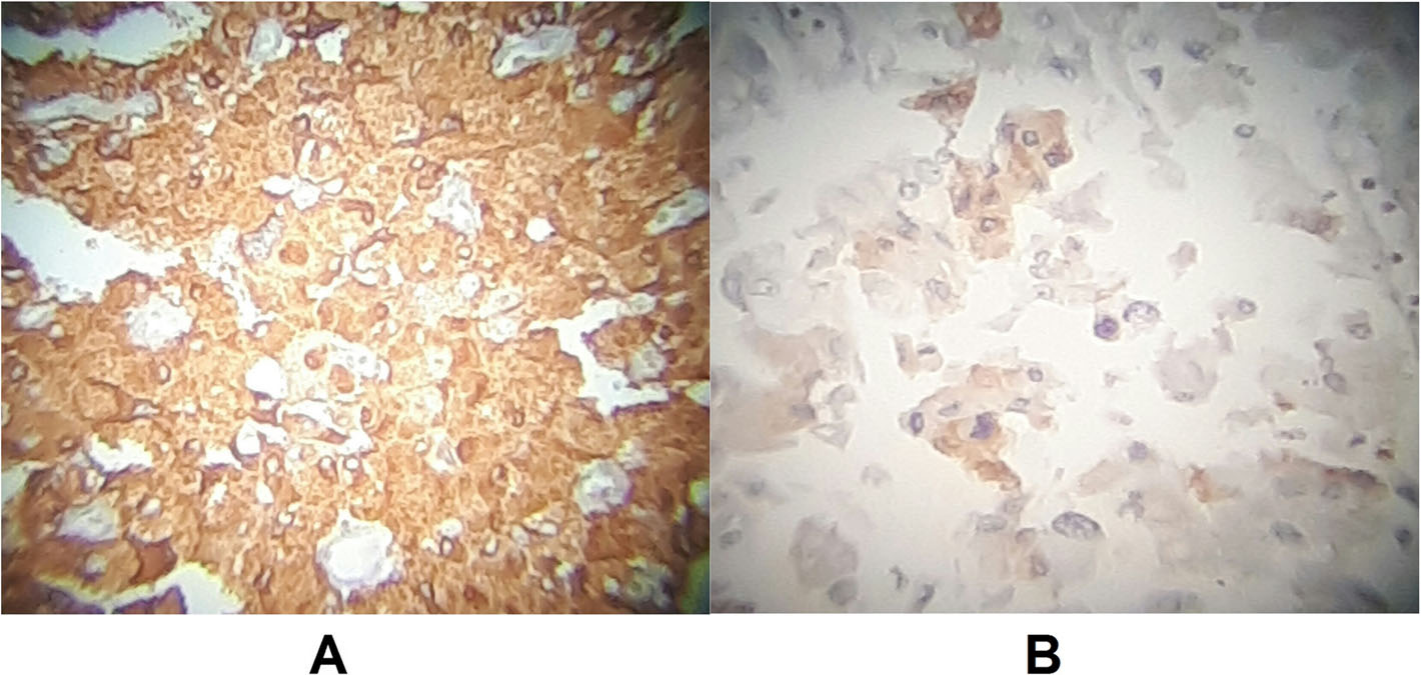
**a** Chromogranin marker: Diffusely Positive. **b** Synaptophysin marker: Focally Positive.

**Fig. 10:**
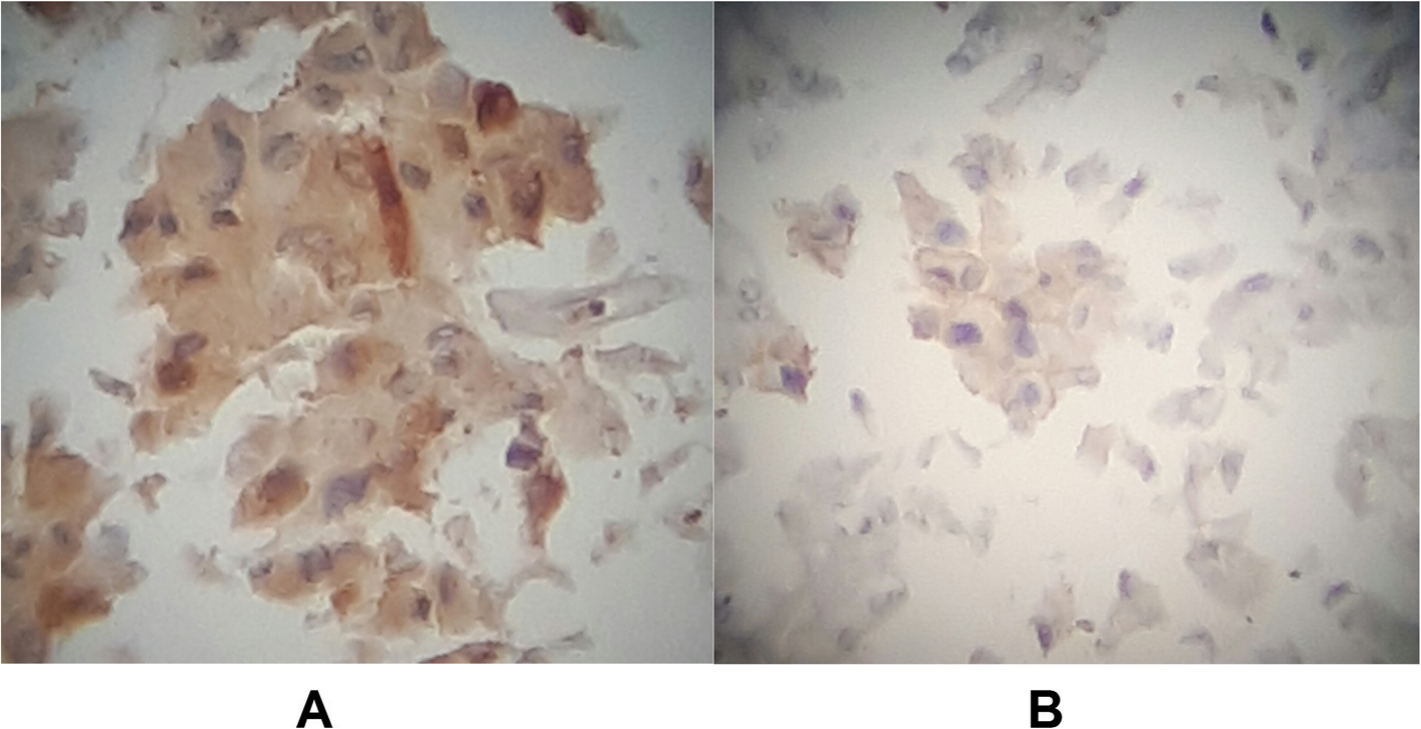
**a** - NSE marker: Focally Positive. **b** CD56 marker: Focally Positive.

**Fig. 11:**
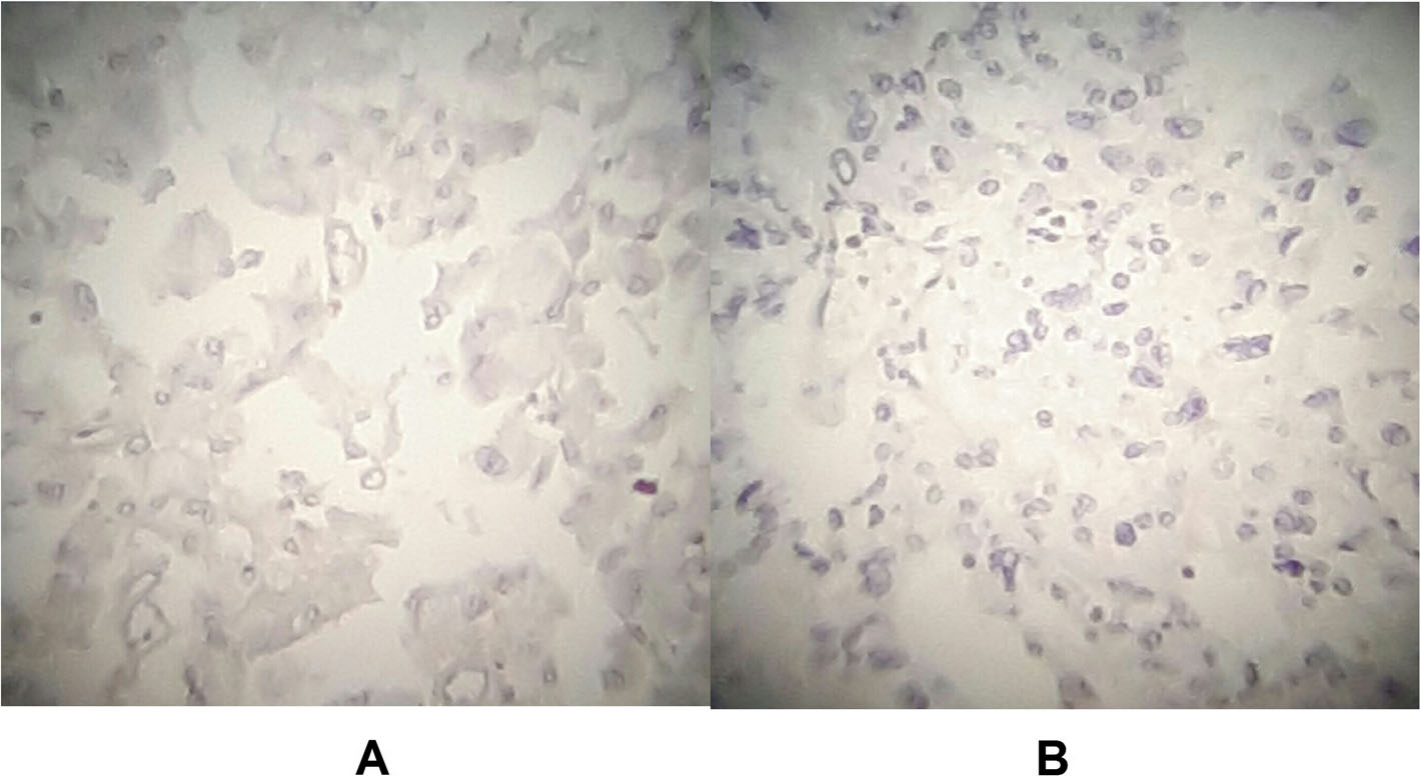
**a** S100 marker: Negative. **b** CK marker: Negative.

**Fig. 12:**
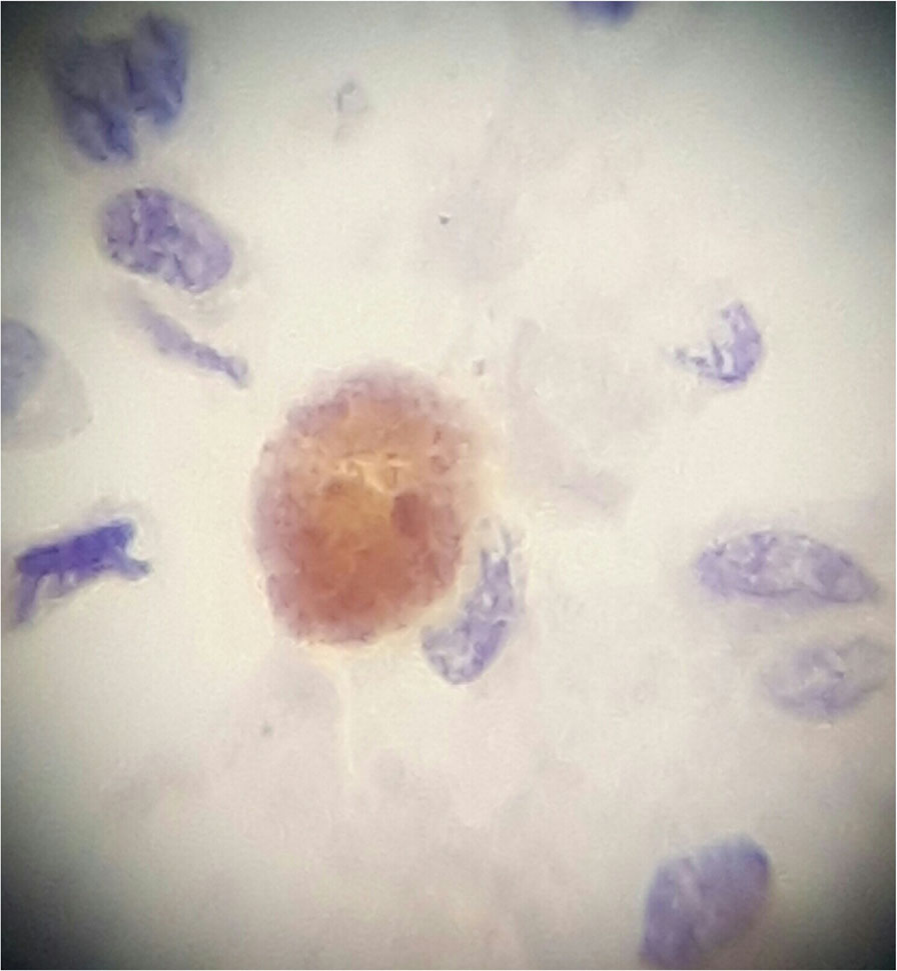
Ki67 marker: Positive for less than ~ 2–3% (low mitotic index).

Preoperative assessment revealed controlled vital signs. During the induction of general anaesthesia and intubation, all vital signs remained normal. When midline incision was preformed, BP and pulse started shooting up (220\130–250\145) mmHg and (120–135) bpm respectively. To control hypertension, deepening the anaesthesia was performed by administration of sedative and analgesic drugs, in addition to vasodilator drugs. The decision was taken to perform minimal manipulation mass excision. At first, the mass appeared to be retroperitoneal adherent to the 4th segment of the duodenum and Treitz ligament with hypervascularized surface and strict adherence posteriorly (Fig. 4). The mass showed neovascularisation from the duodeno-jejunal junction with dense adhesion with the 4th segment of the duodenum without invasion (Fig. 5). Thereafter, the mass was found to be located paravertebral between the aorta and the left kidney and separated from the left kidney and the left adrenal gland. During careful dissection of the mass posteriorly of the aorta and the fatty tissue of the left kidney (Fig. 6), BP and pulse were around (180\110–240\135) mmHg and (130–145) bpm respectively. When the mass main draining vein was ligated, BP dropped dramatically to (50\30) mmHg, so high amounts of crystalloid and vasoconstrictive agents were applied. Due to the mass invasion, an injury of the left renal vein occurred and repair of the vein was carried out. 2000 ml of blood lost and six blood units were transfused. The patient was observed in intensive care unit for 24 h and her vital signs stayed stable. Postoperative course was uneventful. The patient was discharged home on postoperative day six. The histopathology study raised a suspicion of paraganglioma (Figs 7, 8). The immunohistochemical staining studies were positive for neuroendocrine markers chromogranin, synaptophysin, NSE and CD56 (Figs. 9, 10). S100 marker and CK were negative (Fig. 11) and Ki67 marker showed low mitotic index (Fig. 12). Lastly, the diagnosis of paraganglioma was confirmed. All medications were stopped. After 3 months, her follow up was normal. Written informed consent was obtained from the patient.

## Discussion and conclusion

PPGLs are divided according to 2017 WHO classification of adrenal tumors into:

i. PCCs which originate from the medulla of the adrenal gland.
ii. PGLs arise from extra-adrenal paraganglia of the autonomic nervous system [2].

PGLs are divided according to their site and hormonal secretion into:

i. Head and neck parasympathetic PGLs which don’t secrete catecholamine.
ii. Paravertebral sympathetic PGLs of thorax, abdomen and pelvis (85% arise below the diaphragm) that secrete norepinephrine mainly [2, 5].

PGLs are classified as functioning and non-functioning tumors according to their hormonal activity. Functioning PGLs are manifested by the classic triad of headache, palpitation and diaphoresis in only about (24%) of cases. Hypertension is found to be absent (13%), persistent (29%) and paroxysmal (48%) [1]. PGLs could be incidentally found during abdominal imaging in 49% of cases. Our patient did not have the specific symptoms of catecholamine hypersecretion as hypertension and diaphoresis. Occasionally, the unspecific clinical findings of the patient with PGL (psychiatric disorders, anxiety) could make the diagnosis difficult (as in our case) [5]. In C.T scan imaging, PGL and GIST may have a similar radiographic appearance [5]. In case of the presence of unspecific symptoms and the absence of biopsy, PGL may be misdiagnosed as GIST [5].

Duodenal gangliocytic paraganglioma (DGP) is one of the differential diagnoses of duodenal GIST. DGP is a rare nonchromaffin neuroendocrine neoplasm which lacks the ability to secrete catecholamine [6–8]. Both of DGPs and GISTs are located in the submucosal layer, so endoscopic biopsy is usually not exclusive. Abdominal imaging study is unlikely to determine the accurate diagnosis [7]. Precise diagnosis is improbable without the immunohistochemical analysis [6–8].

If PGL is suspected, fractionated plasma metanephrines analysis is the test of choice [9]. The sensitivity of fractionated plasma metanephrines and urinary total metanephrines and catecholamines is (97%) and (90%) respectively [9]. Unfortunately, fractionated plasma metanephrines test is unavailable in our institute. If assay error is excluded, urinary total metanephrines could be false negative (as in our case), in episodically secreting tumors when measured between paroxysmal attacks. In such a case, it is better to do the test during or soon after symptomatic crisis [5, 10]. Biochemically silent PGL in patients with SDHB mutation is a potential other cause for normal plasma or urinary fractionated metanephrines. The absence of tyrosine hydroxylase in such tumors can lead to a defective of catecholamine synthesis [11]. Consequently, such tumors with SDHB mutation can increase in size and manifest with symptoms of surrounding organ compression rather than the classic symptoms of catecholamine hypersecretion. Hence, screening for these tumors should not be limited to catecholamine hypersecretion tests [11].

In our patient, there was no familial history to point to a hereditary reason.

When PLG is still clinically suspected inspite of negative investigations, 123 I-metaiodobenzylguanidine (MIBG) scintigraphy is recommended to detect the tumor (unavailable in our institute) [10, 12]. Fine needle aspiration cytology shouldn’t be performed because of the fear of stimulating hypertension crisis [3].

Obtaining full consideration to the patient’s history and the clinical findings; and subsequently, requesting the proper diagnostic investigations (e.g. fractionated plasma metanephrines or 123 I MIBG scintigraphy in case of normal hormonal tests) could help in the distinction between PGL and other retroperitoneal mass as GIST.

Laparoscopic resection for small non-invasive tumor, otherwise laparotomy for large tumor, is the preferred curative therapy for localized PPGL after adequate preoperative medical preparation [12]. A new recommendation is to prepare all patients with elevated metanephrines or catecholamines, regardless of symptoms, by administration of preoperative alpha-adrenoceptor blockade (phenoxybenzamine) for 7–14 days with or without combination by Beta adrenoceptor blockade [2, 12].

Preoperative hydration and liberal salt intake are necessary to reverse catecholamine-induced blood volume contraction and to avoid hypotension after tumor resection [5, 12].

All PPGLs have a metastatic potential to liver, bones and lymph nodes. Depending on the genotype, metastasis occurs in approximately (10–20%) of PCC and about (50%) in PGL [4]. Therefore, lifelong annual follow-up in PGL is recommended [2, 4]. As 24-h urine metanephrine and normetanephrine were normal in our large sized PGL, SDHB mutation should be assessed. Follow-ups by imaging studies seem warranted and should not be limited to biochemical tests of catecholamine excess.

In case of slow growing metastasis appears on 123 I-MIBG scintigraphy, 131 I-MIBG is recommended as the first-line of treatment. In case of rapid progressing metastasis, chemotherapy (cyclophosphamide, vincristine and dacarbazine) is considered as a palliative therapy [2].

Some of the limitations of our study are that fractionated plasma metanephrines and gene mutations were not assessed.

PLG is life threatening disease and should always be considered as a differential diagnosis of asymptomatic retroperitoneal mass. Due to the dangerous consequences of PGLs, we strongly recommend to evaluate and treat such cases at institutes with adequate expertise.

## Data Availability

Data sharing is not applicable to this article, as no datasets were generated or analyzed during the current study.

## Abbreviations

PCC: Pheochromocytoma
PGL: Paraganglioma
PPGL: Pheochromocytoma and paraganglioma
GIST: Gastrointestinal stromal tumor
Bpm: Beat per minute
BP: Blood pressure
C.T: Computed tomography
DGP: Duodenal gangliocytic paraganglioma
MIBG: Metaiodobenzylguanidine
